# Effects of Seawater Environment on the Degradation of GFRP Composites by Molecular Dynamics Method

**DOI:** 10.3390/polym14142804

**Published:** 2022-07-09

**Authors:** Xiuli Zhang, Zongcai Deng

**Affiliations:** 1 Beijing Building Materials Science Academy, Beijing 100041, China; 2 Faculty of Architecture, Civil and Transportation Engineering, Beijing University of Technology, Beijing 100124, China; dengzc@bjut.edu.cn

**Keywords:** GFRP, degradation, seawater, molecular dynamics, durability

## Abstract

Glass fiber-reinforced polymer (GFRP) composites are promising composites often utilized in coastal infrastructure or used as an alternative to steel reinforcement in seawater sea sand concrete due to their excellent corrosion resistance. Understanding the degradation mechanism of GFRP in corrosion environments is significant for improving the long-term performance of GFRP materials. This paper presented the influences of seawater content and temperature on the properties of GFRP composites using the molecular dynamics method. The simulation results were validated by existing experiments on mechanical properties, interlaminar strength, and microstructures of an accelerated aging test of GFRP. The calculation results indicated that when seawater content of the matrix increased from 0% to 9.09% at 298 K, Young’s modulus, shear modulus, and bulk modulus decreased 46.72%, 53.46%, and 41.75%, respectively. The binding energy of GFRP composites with seawater content of 2.15% at 353 K was 26.46% lower than that of unconditioned GFRP at 298 K. It revealed that the higher seawater content and temperature accelerated the degradation of the GFRP composites. The investigation provided a comprehensive understanding of the degradation mechanism of GFRP in seawater environments and provided a basis for the durability design of GFRP composites.

## 1. Introduction

There is a rising demand for coastal infrastructure and an expanded application of seawater sea sand due to the resource shortage of fresh water and river sand. Fiber-reinforced polymer (FRP) composites became promising composites used in coastal engineering structures or seawater and sea sand concrete structures for their attractive features of being lightweight, high strength, easy to install, and ultraviolet, and especially for their corrosion resistance [1,2,3,4]. They could be used to rehabilitate and retrofit the existing structures or utilized as an alternative to the steel bars in the new engineering structures.

FRP composites are manufactured in different forms, such as sheets, laminates, and bars. In general, FRP composites are made up of one of three types of fibers (glass, carbon, or basalt), and the composites are called GFRP, CFRP, and BFRP, respectively [2]. Comparing the performance of these three FRP composites, CFRP has a higher cost and lower elongation at break but has superior mechanical (stiffness and strength), fatigue, creep, and corrosion resistance properties. GFRP and BFRP have relatively lower costs and higher fracture toughness. However, the durability of GFRP and BFRP is weaker, especially in typical temperature/load coupled environments [4,5,6,7].

In the three FRP composites, the high cost is the main obstacle to the application of CFRP in civil engineering, while GFRP is widely used in civil engineering due to its relatively low cost, considerable durability and mechanical properties [8,9,10,11].

Understanding the influence of seawater environments on the durability of GFRP is important since the structures are usually exposed to marine environments (such as oceans, ports, and bridges). The durability of the GFRP was related to the performance of fiber, matrix, and fiber/matrix interface in the composites [12].

The resin matrix in the GFRP composite bonds the fibers together and spreads the load applied to the composite between each individual fiber. The resin matrix also protects the fibers from erosion in severe environments. The properties of the resin system in GFRP were critical for the mechanical properties and durability of GFRP [13]. Epoxy resins are widely used in various industrial applications [14,15] and exhibited good resistance to UV irradiation and moisture [16]. It was widely used in FRP composites for its excellent physical and mechanical properties [13].

The epoxy resin matrix could absorb moisture from 1% to 7% by weight [3]. When the FRP composites were exposed to seawater, the Na^+^, Cl^−^, and water molecules penetrated the composites [17,18]. Mourad et al. [19] carried out a study on the durability of GFRP composites in seawater environments. After immersing for 1 year, the seawater absorption of the glass/epoxy composites by weight was 2.5% at room temperature and 5% at 65 °C, and the reduction in tensile strength was 0.8% at room temperature and 6% at 65 °C. The higher temperature accelerated the absorption of water molecules and degradation of the tensile strength of the composites. Silva et al. [18] investigated the durability of the GFRP laminate in a salt solution. The results indicated that after immersing for 750 h, the Na^+^, Cl^−^, and water molecules reached the fibers. The value of maximum moisture absorption increased with the increasing temperature. After immersing in the salt solution for 2500 h, the tensile strength retentions of the GFRP laminate at 35 °C, 50 °C, and 65 °C were 84%, 70%, and 61%, respectively. Due to the absorption of seawater, the matrix in GFRP composites swelled and plasticized, which caused microcracking and fracturing [20]. The microcracks accelerated the diffusion of seawater into the GFRP composites. The different volumetric expansion of components in the GFRP composites caused internal stress that reduced the bond between fibers and polymers [21]. The degradation of the matrix and the fiber/matrix interface resulted in the degradation of the mechanical properties of the GFRP composites.

Many researchers conducted experiments to investigate the effect of seawater environments on the mechanical performance of the GFRP composites, such as tensile strength, compressive strength, shear strength, and elastic modulus. The seawater environment was usually simulated by salt solution [20,22,23]. The SEM results showed that the fiber/matrix interface was seriously damaged in seawater environments, and after the aging time of 90 days, the tensile strength retention was approximately 70% [17]. Tannous and Saadatmanesh [20] investigated the durability of the alkali-resistant (AR) GFRP bars immersed in two kinds of salt solutions. It showed that the degradations of the tensile strength of the composites immersed in the solutions for 6 months were 22.7% and 25.1%, respectively. The experiment conducted by Zhang and Ou [24] indicated that the GFRP bars lost 4.36% and 6.83% of their original tensile strength after immersing in the salt solution at 60 °C for 28 days and 56 days, respectively. Kim et al. [23] conducted experiments on the durability of the GFRP bars. After immersing in the salt solution for 132 days at 25 ℃, 40 °C, and 80 °C, the tensile strength retentions of the specimen G1 were 86.4%, 87.4%, and 80.7%, and that of the specimen G2 were 80.8%, 82.5%, and 42.6%. Benmokrane et al. [25] performed a study on the durability of the solid GFRP rods. After exposure to the saline solution for 5000 h, the retentions of the tensile strength, elastic modulus, and ultimate strain were 95.1%, 96.5%, and 88.6%. The compressive properties of GFRP bars exposed to the salt solution were investigated. After exposure to the salt solution at 40 °C and 80 °C for 90 days, the compressive strength retentions of GFRP bars without sustained stress were 83% and 67%, respectively [26]. After exposure to the salt solution at 60 °C for 42 days and at 25 °C for 100 days, the compressive strength retentions of the GFRP bars without sustained stress were 77.8% and 89% [27]. The interfacial shear properties of GFRP were also studied. After immersing in the chloride environment at 80 °C for 90 days, the interfacial shear strength retentions of the 2 types of GFRP bars were 87.5% and 79.9%, respectively [23]. After exposure to the NaCl solution at 60 °C for 50 days, the retention of the interlaminar shear strength was 80.5% [28].

**Figure 1:**
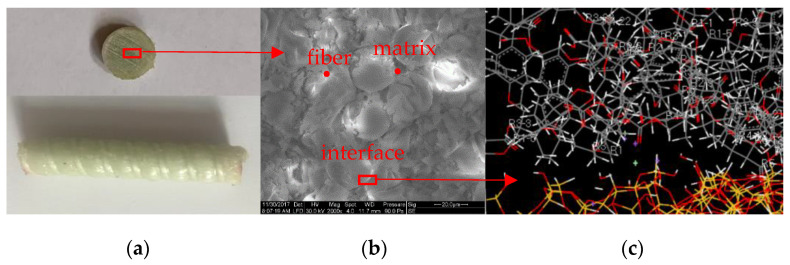
GFRP composites in multiscale. (**a**) Macroscale. (**b**) Microscale. (**c**) Nanoscale.

Though the durability of the GFRP composites has been studied by various experiments and techniques in macroscale and microscale shown in Figure 1a,b, such as compression test, tension test, short beam test, and scanning electron microscope (SEM), the interfaces of materials were usually of the nanometer length scale shown in Figure 1c. The degradation of the GFRP composites occurred at a molecular level; however, using the traditional research methods proved difficult for providing a thorough understanding of the degradation.

Molecular dynamic (MD) simulation could show the details of molecular motion and molecular distribution, which were critical to the performance of the GFRP composites. Some research has been carried out on the properties of the matrix and interface in the composites using the MD method. Nouri and Ziaei-Rad [29] studied the optimal ratio of resin to cure agent in the polymer by analyzing the density and bulk modulus of the polymer. Deng et al. [30] investigated the interface properties of polycarbonate and silanes by calculating the work of adhesion. The results revealed that the composites with a higher work of adhesion may not have a higher interfacial toughness. Subramanian et al. [31] investigated the defects of the fiber surface on the interfacial energy and cohesive behavior of interphases in carbon fiber reinforced carbon nanotubes/epoxy composites. A few researchers investigated the effect of corrosion environments on the properties of the composites. Zaminpayma and Mirabbaszadeh [32] studied the effect of temperature on the CNT/polymers interface. The interface property of CFRP-wood composites degraded with the duration in moisture conditions or higher temperatures [33,34]. The interfacial adhesion between carbon fiber and epoxy resin in the composites decreased due to the water ingress [35]. The corrosion resistance of GFRP composites in seawater environments was related not only to the properties of the components (fibers and matrix) but also to the integrity of the fiber/matrix interface while aging. The matrix played an important role in bonding the fibers together [36], and the interface transferred the loads between fibers. The matrix and the interface were susceptible to the temperature and corrosive environment [22,37]. Although these investigation methods are effective, there was inadequate research at an atomic level on the degradation mechanism of GFRP composites in seawater. Tests at such a length scale are expensive and difficult to implement. Thus, the molecular dynamics simulation becomes a feasible method to reveal the degradation mechanism of the resin matrix and fiber/resin interface [38].

This paper aimed to investigate the effect of seawater environments on the matrix and fiber/matrix interface properties in the GFRP composites by using MD simulation. The existing research showed that the durability of the GFRP in salt solution was related to chloride concentration and immersing temperature [20,26]. The numerical models of matrix and fiber/matrix were constructed to investigate the effect of seawater content and temperature on the performance of the GFRP composites. The physical and mechanical properties of the resin matrix were studied. The interfacial properties were determined by calculating the interaction energy of the fiber/matrix interface, mean square displacement, and relative concentration of resin matrix on the fiber surface. The accelerated aging test of GFRP composites was conducted to validate the MD simulation results.

## 2. Computational Method

In the MD computational process, the COMPASSIIforce field was employed, and the cutoff distance was 9.5 Å. The temperature and pressure were controlled using the Andersen and Berendsen method. The geometry optimization of models was performed by a smart algorithm.

### 2.1. The Epoxy Resin Model in Seawater Environment

**Figure 2:**
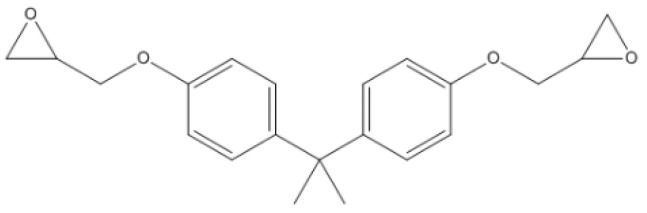
Monomer molecular module of DGEBA.

**Figure 3:**
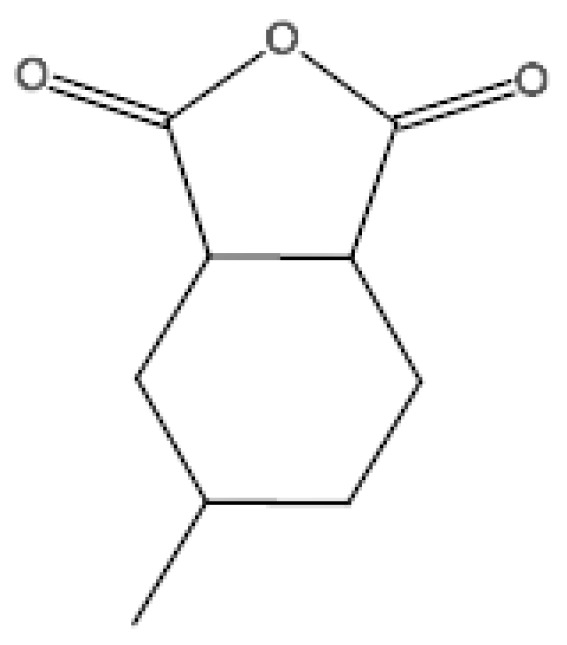
Monomer molecular module of MeHHPA.

In the epoxy resin model, diglycidyl ether of bisphenol A (DGEBA) was used as the base epoxy resin, and hexahydro-4-methylphthalic anhydride (MeHHPA) was used as the curing agent. The structures of DGEBA and MeHHPA are shown in Figure 2 and Figure 3. The amorphous cell was composed of 22 DGEBA and 44 MeHHPA and had an initial density of 1.2 g/cm^3^. The dimensions of the cell were 27.41 Å × 27.41 Å × 27.41 Å. The cell was minimized by 2000 iterations of geometry optimization. After that, it was equilibrated at 298 K (25 °C) for 50 ps in canonical ensemble (NVT). Then it was equilibrated at 298 K (25 °C) and 1 atm for 50 ps in an isothermal-isobaric (NPT) ensemble. The time step was 1 fs.

**Figure 4:**
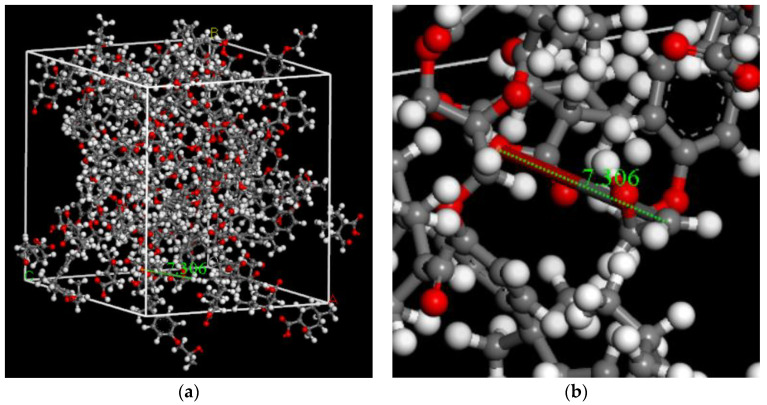
The crosslinked epoxy resin model. (**a**) The model after curing process. (**b**) The new bonds in crosslinked model.

The curing process of DGEBA and MEHHPA was performed using a Perl script. The new bonds were created between reactive atoms when they were within the cutoff distances. The cutoff distance was 3.5–7.5 Å with an increment value of 1 Å. After reaching the maximum cutoff distance or if no new bond was formed, the crosslinking process was stopped. At last, the polymer network was equilibrated at 298 K (25 °C) for 500 ps in the NVT ensemble and then at 298 K (25 °C) and 1 atm for 500 ps in the NPT ensemble. The epoxy resin model after curing process was shown in Figure 4a and the new bonds in crosslinked model were shown in Figure 4b.

**Figure 5:**
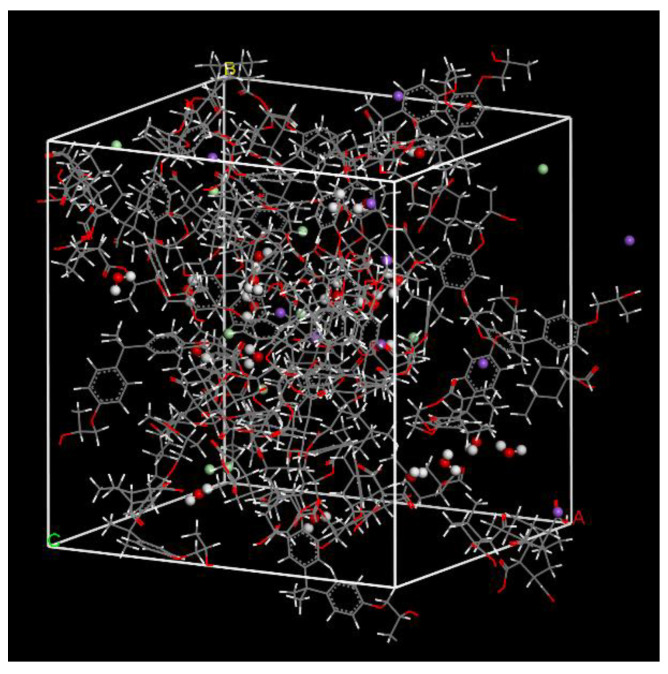
The resin matrix models in a seawater environment.

To simulate a seawater environment, the Na^+^, Cl^−^, and water molecules were added into the crosslinked epoxy system in the ratio of 1:1:2, as shown in Figure 5. A total of 3 epoxy resin models with 3.03–9.09% of seawater were constructed to study the seawater environment on the properties of epoxy resin of GFRP composites. Each model performed the geometry optimization and dynamics procedure at 298 K (25 °C).

### 2.2. The Fiber/Matrix Interface Model in Seawater Environment

To study the seawater environment on the properties of the fiber/matrix interface of the FRP composites, four fiber/matrix interface models of different seawater contents were constructed.

**Figure 6:**
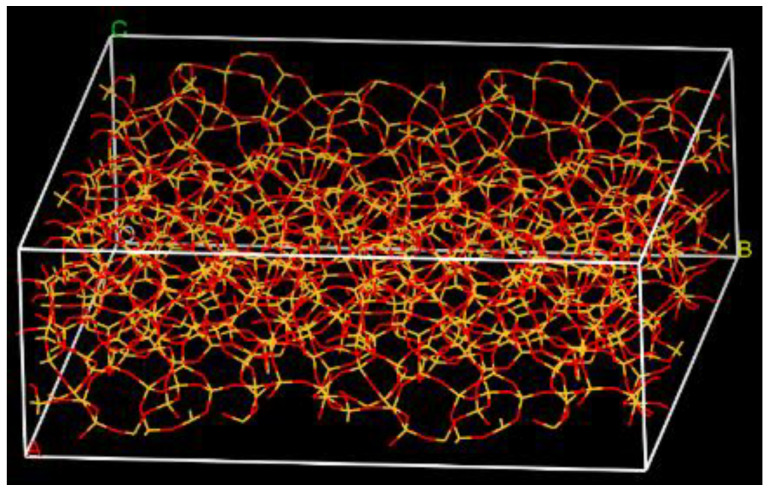
The glass fiber model.

The main component of the glass fiber was SiO_2_ [17]. Thus, the glass fiber system was simplified to a SiO_2_ structure [39]. The initial structure dimension was 21.394 Å × 21.394 Å × 21.394 Å, and the density was 2.2 g/cm^3^. The structure was cleaved to form a surface for the fiber/matrix interface of the composite. The cleave plane was (0, 0, −1), and the cleave thickness was 15 Å. To make the surface model closer to the actual structure, the model was increased to a new dimension of 42.789 Å × 42.789 Å × 14.895 Å, shown in Figure 6, resulting in a larger interface between the fiber and the matrix. The enlarged surface was changed to a three-dimensional structure by building a vacuum slab. Then the glass fiber model performed an equilibration process including 2000 iterations of geometric optimization, 50 ps NVT dynamics at 298 K, and 10 ps NPT dynamics at 298 K (25 °C) and 1 atm.

**Figure 7:**
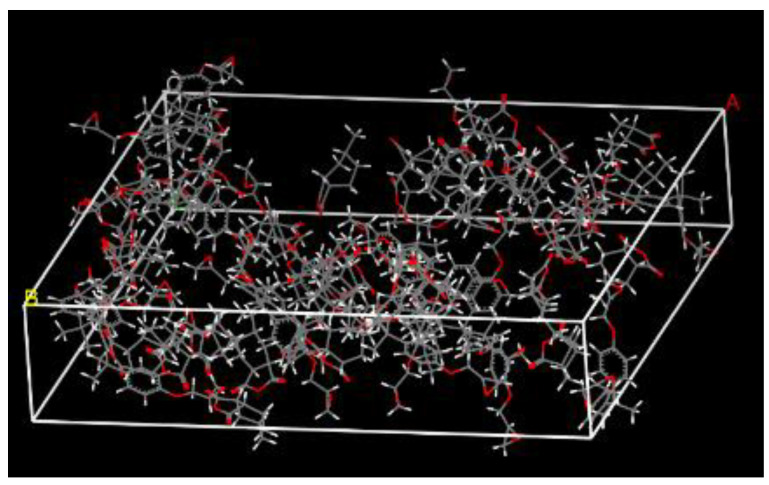
The resin matrix model.

The epoxy resin matrix in the fiber/matrix interface model consisted of 18 DGEBA and 36 MeHHPA molecules. The cell dimension of the amorphous, shown in Figure 7, was 42.789 Å × 42.789 Å × 9.207 Å, which matched the size of the glass fiber surface. The density was 1.2 g/cm^3^. Then the model performed the same equilibration process as the fiber surface. The uncrosslinked epoxy resin model is shown in Figure 7.

**Figure 8:**
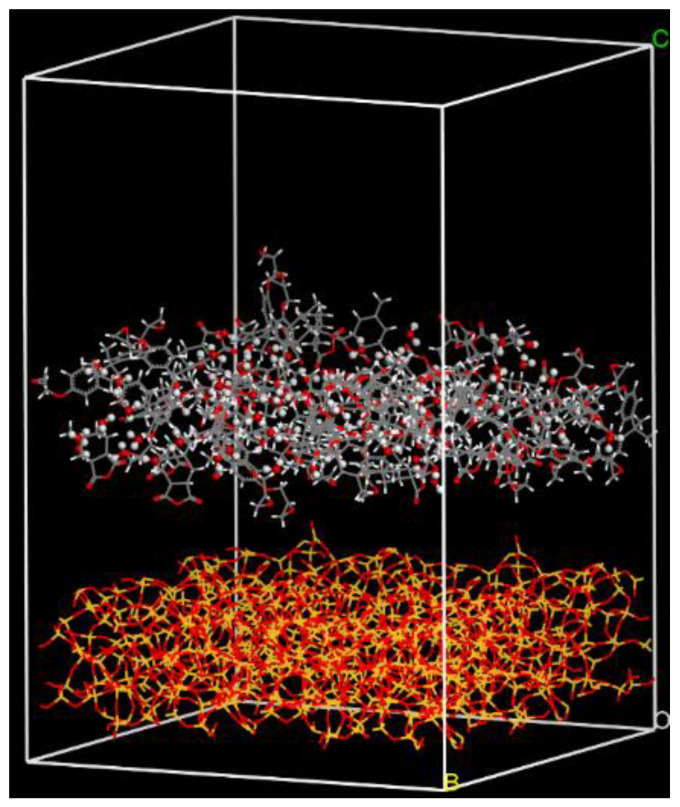
The fiber/matrix interface model.

The interface model was constructed by adding the matrix layer to the surface of the fiber, as shown in Figure 8. The fiber surface had a vacuum slab of 0 Å. The matrix layer had a vacuum slab of 20 Å. Then the interface model experienced the same crosslink procedure as the epoxy resin model performed.

**Figure 9:**
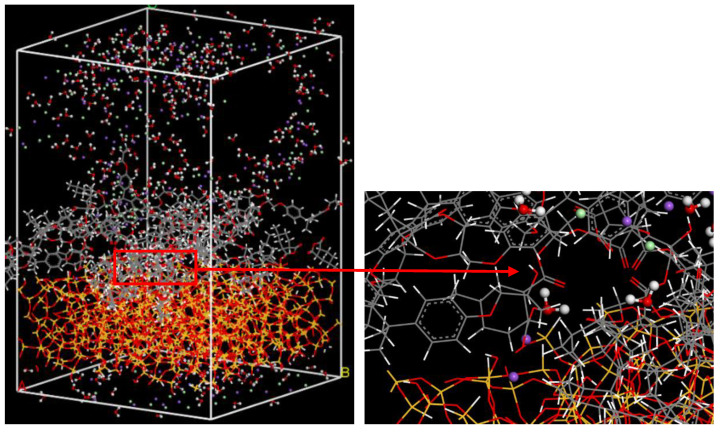
The fiber/matrix interface model in the seawater environment.

To study the effect of seawater on the fiber/matrix interface of GFRP composites, the Na^+^, Cl^−^, and water molecules in the ratio of 1:1:2 were packed into the composites, as shown in Figure 9. The seawater immersed in the vacuum of the composites model was not counted when calculating the seawater content by weight. Only the molecules and irons between the top surface of the epoxy layer and the bottom surface of fiber were included in the calculation of seawater content. Seven interface models were constructed to investigate the effect of seawater content and temperature on the interface. The model code was made up of letters and numbers. The letters M and S stood for the dry environment and seawater environment. The first and second numbers stood for the seawater content and temperature. For example, S2.15%-298 K represented that the seawater content of the model was 2.15%, and the temperature of the model was 298 K (25 °C). In the models, the purple balls were Na^+^ and the green balls were Cl^−^.

## 3. Results and Discussion

### 3.1. The Properties of Epoxy Resin System

#### 3.1.1. The Plasticization of Epoxy Resin System

**Figure 10:**
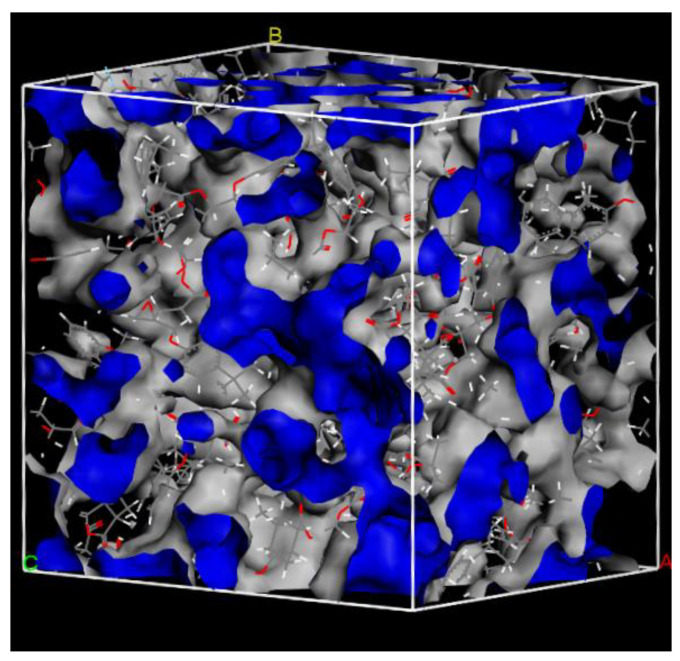
Schematic diagram of free volume of the resin matrix in the seawater environment.

The free volume of the system containing seawater is shown in Figure 10. The surface of the free volume is marked in gray color. Free volume is the space between molecules, and it is the motion space of the molecules in the system. Free volume could reflect the compactness of materials. The free volume of the systems containing seawater was calculated as shown in Table 1.

In the salt solution, ions penetrated along with the water molecules into the composites, which caused damage to the matrix [17]. The results showed that when the Na^+^, Cl^−^, and water molecules entered the resin matrix, the volume expansion rate increased obviously, inducing the swelling of the resin matrix. As the seawater content increased, the fractional free volume increased obviously at the initial stage and then decreased slowly, while the density had an opposite trend, which may be due to the existence of the Na^+^ and Cl^−^ in seawater.

#### 3.1.2. The Mechanical Properties of Epoxy Resin System

The mechanical properties of the epoxy resin system were calculated using the constant strain method. The maximum strain amplitude was 0.003. The models with the seawater weight containing 0%, 3.03%, 6.06%, and 9.09% were constructed to calculate Young’s modulus, shear modulus, bulk modulus, and Poisson’s ratio. The calculation results are shown in Table 2.

Some researchers conducted experiments on the mechanical properties of epoxy resin. The tensile elastic modulus and Poisson’s ratio were 3.1 GPa and 0.3 [40]. The Young’s modulus, shear modulus, bulk modulus, and Poisson’s ratio were 4.15–4.17 GPa, 1.33–1.75 GPa, and 5.01–5.80 GPa [41]. The compressive elastic modulus was 4.83 GPa [42]. The models were validated considering the test data. Wang et al. revealed that the plasticization of the resin matrix led to the appearance of micropores and microcracks [43].

The calculation results indicated that when seawater content increased from 0% to 3.03%, 6.06%, and 9.09%, Young’s modulus decreased 23.36%, 34.42%, and 46.72%, the shear modulus decreased 25.36%, 37.33%, and 53.46%, and the bulk modulus decreased 22.33%, 30.34%, and 41.75%; however, the Poisson’s ratio did not significantly change.

Hu et al. investigated the properties of two types of epoxy in seawater. The results indicated that after immersing for 150 days, the elastic modulus of the resin of type B decreased by 24%, which verified the accuracy of this model [44].

### 3.2. The Properties of Fiber/Matrix Interface of the Composites

#### 3.2.1. The Interaction Energy between Fiber and Epoxy Resin Matrix

The interaction intensity between the fiber and epoxy resin matrix could be reflected by the interaction energy as follow:

△*E* = *E* − (*E*_1_ + *E*_2_) (1)

where △*E* was the interaction energy between the fiber and epoxy resin matrix. *E* was the total energy of the composites, and *E*_1_ was the energy of the resin matrix without fiber. *E*_2_ was the energy of the fiber without the resin matrix.

Binding energy was the negative value of the interface energy. The higher the binding energy was, the more work was needed to separate the fiber and epoxy resin.

**Figure 11:**
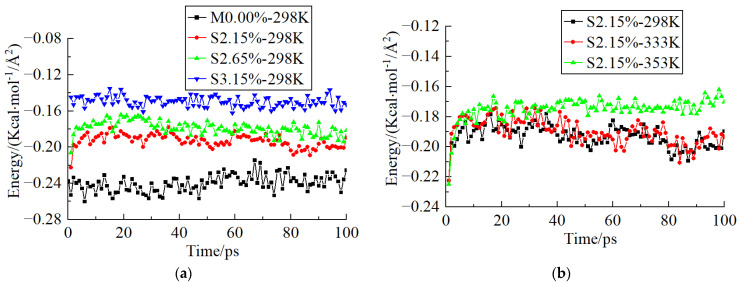
Interface energy of systems. (**a**) Effect of Seawater Content. (**b**) Effect of Temperature.

The fiber/resin matrix interface model performed a 100 ps NVT MD simulation followed by a 100 ps NPT MD simulation. The step time was 1 fs. The interface energy in the equilibration process is shown in Figure 11. The value of the interaction energy was negative, which indicated that the epoxy resin was attracted to the fiber surface. Figure 11 shows the effect of seawater content and temperature on the interface energy of the systems.

As shown in Figure 11a, when the systems were at 298 K (25 ℃), the interface energy of the system increased obviously with the increasing seawater content. Figure 11b showed that when the seawater content was 2.15%, the interface energy of the system at 298 K (25 °C) and 333 K (60 °C) were similar, and they were obviously higher than that at 353 K (80 °C).

The calculation results shown in Table 3 are the average value of the last 50 ps in the simulation process. The system conditioned in the dry environment had the lowest interface energy.

As shown in Table 3, the interface energy increased with the increasing seawater content or temperature. The higher the interface energy, the lower the interface stability. Some studies have explored the interface properties of GFRP composites. Wang et al. investigated the effect of fiber surface functionalization on shear behavior at the carbon fiber/epoxy interface by molecular dynamics analysis and experiment. The results indicated that the shear energy has a linear relationship with the shear strength of FRP composites [38]. The experiment conducted by Bian et.al showed that after immersing in seawater for 14 days, 28 days, and 42 days, the interlaminar shear strength of GFRP composites with a fiber volume fraction of 44% decreased 46%, 50%, and 50%, respectively. The interlaminar shear strength is affected by the adhesive interface strength between the fiber and the resin matrix. The shear strength decreased due to the debonding of the fiber/matrix interface [11]. The experiment results agreed well with the simulation results.

#### 3.2.2. The Diffusion of Epoxy Resin on the Fiber Surface

The motion of the epoxy resin chains could be reflected by the mean square displacement (MSD), which was defined as follows:

(2) $$\mathrm{MSD}(t)=\frac{1}{3N}\sum_{i=0}^{N-1}\langle {\left|r_{i}(t)-r_{i}(0)\right|}^{2}\rangle$$

where *N* was the total number of atoms of epoxy resin, *r*_i_(*t*) and *r*_i_(0) were the positions of the atom center of mass at time *t* and 0, respectively, and the angular bracket represented averaging over all choices of time origins.

**Figure 12:**
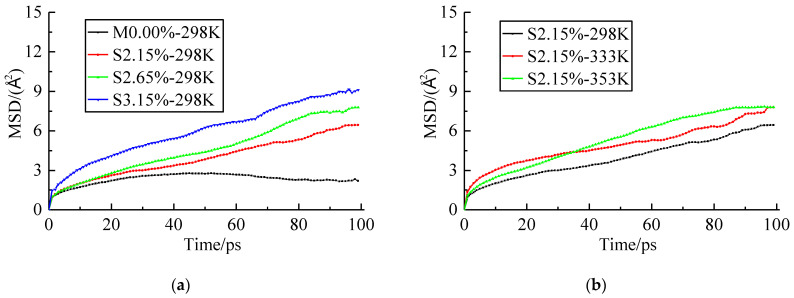
MSD of the resin matrix. (**a**) Effect of Seawater Content. (**b**) Effect of Temperature.

In order to analyze the effect of seawater content and temperature on the MSD, the MSD curves of the resin matrix are shown in Figure 12.

As seen in Figure 12, the MSD curves of the epoxy resin with higher seawater content were higher than those with lower seawater content when the systems were at the same temperature. It demonstrated that the higher seawater content accelerated the motion of the epoxy resin network chains. The MSD curves of the epoxy resin at a higher temperature were higher than those at a lower temperature when the systems had the same seawater content. It demonstrated that the higher temperature or seawater content accelerated the motions of epoxy resin network chains. Thus, the properties of the composites deteriorated.

#### 3.2.3. The Distribution of Epoxy Resin in the Composites

The distribution of epoxy resin on fiber surfaces in composites could be illustrated by the relative concentration. The concentration profile was the atom density as the function of the distance from the origin of a given direction. The relative concentration was calculated as follows.

(3) $$C=\frac{N_{\mathrm{slab}}}{V_{\mathrm{slab}}}/\frac{N_{\mathrm{tot}}}{V_{\mathrm{tot}}}$$

where *N*_slab_ was the number of atoms in the slab, *V*_slab_ was the slab volume, *N*_tot_ was the total number of atoms in the system, and *V*_tot_ was the total volume of the system.

**Figure 13:**
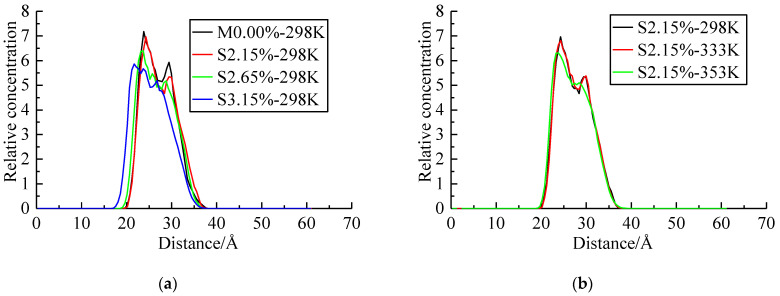
Concentration profiles of the resin matrix. (**a**) Effect of seawater content. (**b**) Effect of temperature.

Figure 13 was plotted to investigate the effect of seawater content and temperature on the relative concentration of epoxy resin. The value of the *x*-axis represented the distance to the bottom of the fiber.

As can be seen in Figure 13, the shapes of relative concentration-distance curves of all the systems were similar. With the increasing seawater content or temperature, the peak relative concentration decreased and the concentration distribution became more uniform.

It was noted that the relative concentration of epoxy resin on the surface of fiber was obviously higher than that inside the epoxy resin matrix, which was due to the attraction effect of fiber on the resin matrix.

As shown in Figure 13a, the peak relative concentration of epoxy resin on the fiber surface of the system with seawater contents of 0.00%, 2.15%, 2.65%, and 3.15% were 7.17, 6.97, 6.40, and 5.87, respectively. The results demonstrated that when the seawater content was higher than 2.15%, the seawater content had a significant effect on the relative concentration. As shown in Figure 13b, the peak relative concentration of epoxy resin on the fiber surface of the system at 298 K (25 °C), 333 K (60 °C), and 353 K (80 °C) were 5.87, 6.81, and 6.3, respectively. When the temperature was higher than 333 K (60 °C), the relative concentration had an obvious decrease. It showed that the higher temperature or higher seawater content reduced the attraction effect of fiber on epoxy resin. It demonstrated that the residual resin matrix that remained on the fiber surface decreased as the seawater content or temperature increased. Thus, the interface properties deteriorated.

**Figure 14:**
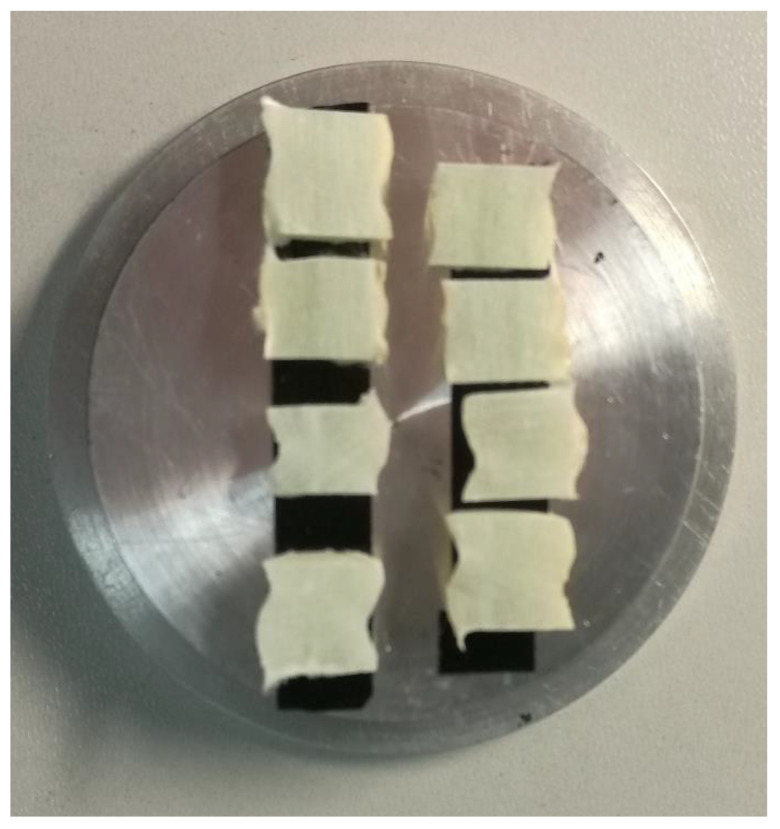
The GFRP samples for SEM.

**Figure 15:**
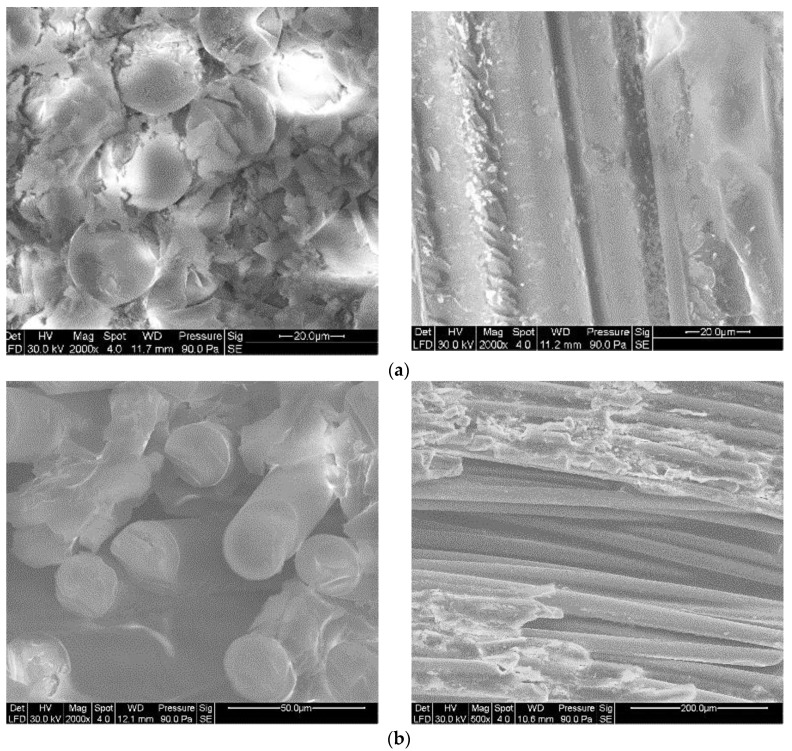

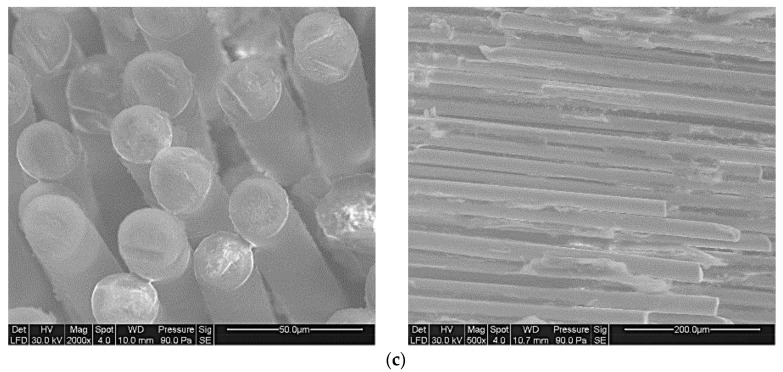
The SEM photographs. (**a**) In Dry Environment at 25 °C. (**b**) In Salt Solution at 25 °C for 90 days. (**c**) In Salt Solution at 80 °C for 90 days.

An accelerated aging test of GFRP composites immersed in a simulated seawater environment was conducted to validate the MD simulation results. The GFRP composites used in the experiment are shown in Figure 1a. The GFRP composites were made of continuous E-glass fibers impregnated in a resin matrix using the pultrusion process. The resin matrix consisted of epoxy resin diglycidyl ether bisphenol A (DGEBA) and a curing agent of methyl hexahydrophthalic anhydride (MeHHPA). The fiber volume fraction was 70%. The diameter of the GFRP bars was 10 mm. The seawater environment was simulated by NaCl solution of 3% by weight. The salt solution was contained in a thermostatically controlled liquid bath which maintained the test sample at a temperature of 25 °C or 80 °C. The samples were exposed to the solution at 25 °C and 80 °C for 90 days, respectively. The conditioned samples are shown in Figure 14. Then the microstructures of samples were analyzed by scanning electron microscopy (SEM). The SEM photographs of the cross-section and longitudinal section of the unconditioned and conditioned samples are shown in Figure 15.

Figure 15 showed that all the fibers in the samples were intact, which indicated that the performance of the glass fibers in a salt environment was stable.

As the immersing time increased, the seawater content increased, so the comparison results of Figure 15a,b show the effect of seawater content on the fiber/matrix interfacial performance. The seawater absorption of GFRP composites increased with the immersing time. The SEM photos revealed that with the increasing seawater content, the resin matrix covering the fibers decreased, inducing the deterioration of the fiber/matrix interfacial performance. The comparison results of Figure 15a,c show that the resin matrix covering the fibers decreased when the immersing temperature increased from room temperature to 353 K (80 °C). The MD simulation results were in accordance with the experiment results that the peak relative concentration decreased with the increasing seawater content or temperature.

## 4. Conclusions

In this study, the influences of seawater content and temperature on the properties of GFRP composites were investigated using the MD simulation method. The physical and mechanical properties of the resin matrix and the fiber/matrix interface energy of GFRP composites, MSD, and relative concentration of the matrix on the fiber surface were analyzed. The following conclusions were drawn:

1. Comparing the simulated mechanical properties of resin matrix to the existing experiment data and the fiber/matrix interface energy to the results of the interlaminar shear strength experiment, the calculation results agreed well with the existing experiment results. The calculation method is validated to be reasonable and effective and can be used to predict the properties of GFRP composites in severe environments.
2. The calculation results of density, volume expansion rate, Young’s modulus, shear modulus, and bulk modulus indicated that as the seawater content increased, the physical and mechanical properties of the resin matrix degraded due to the plasticization. That was one of the main reasons that induced the degradation of the GFRP composites in seawater environments.
3. The simulation results showed that with the increasing seawater content, the binding energy decreased, the motion of the matrix on the fiber surface became faster, and the concentration of the matrix on the fiber surface decreased. The interface performance degraded as the seawater penetrated the GFRP composites. That was another main reason that induced the degradation of the GFRP composites in seawater environments.
4. The higher temperature promoted the water molecular, Na^+^, and Cl^−^ penetrating the GFRP composites, which accelerated the mobility of the polymer chains and promoted the diffusion of seawater into the composites. The higher temperature accelerated the degradation of the composites.
5. The MD simulation method can be used to analyze the degradation of the mechanical properties of GFRP composites in seawater environments and provide a deeper understanding of the degradation mechanism. So, the methods presented in this study could provide a new idea to predict the long-term durability of GFRP composites in complex environments, such as an environment in which alkali and salt work together or an environment coupled with sustained loads.

## Figures and Tables

**Table 1:** Density and volume of resin matrix in the seawater environment.

Seawater Content/%	Density/(g/cm^3^)	Volume/Å^3^	Volume Expansion Rate/%	Fractional Free Volume/%
0.00	1.1548	21,435.4	0.000	15.9835
3.03	1.1239	22,725.0	5.9911	18.7354
6.06	1.1320	23,253.6	8.4469	17.5276
9.09	1.1405	23,768.6	10.8394	17.5613

Note: Fractional free volume was the ratio of free volume and total volume.

**Table 2:** Mechanical properties of resin matrix in the seawater environment.

Seawater Content/%	Young’s Modulus/GPa	Shear Modulus/GPa	Bulk Modulus/GPa	Poisson’s Ratio
0.00	4.88	2.17	4.12	0.35
3.03	3.74	1.62	3.20	0.37
6.06	3.20	1.36	2.87	0.36
9.09	2.60	1.01	2.40	0.36

**Table 3:** The potential energy of the composites and the interaction energy.

Model	*E*/(kcal/mol)	*E*_1_/(kcal/mol)	*E*_2_/(kcal/mol)	Δ*E*/(kcal/mol)	Δ*E*/*A* (kcal/(mol·Å^2^)
M0.00%-298 K	−91,124.73	1954.12	−92,714.94	−399.67	−0.2350
S2.15%-298 K	−91,004.80	2029.04	−92,699.96	−333.88	−0.1961
S2.65%-298 K	−90,898.13	2085.50	−92,675.74	−307.89	−0.1826
S3.15%-298 K	−90,390.54	2259.84	−92,395.17	−255.21	−0.1507
S2.15%-333 K	−90,503.40	2254.88	−92,427.58	−330.70	−0.1942
S2.15%-353 K	−89,918.30	2350.72	−92,268.98	−291.86	−0.1728

Note: *A* was the interface area.

## Data Availability

Not applicable.
